# Investigating the effect of a magnetic field on dose distributions at phantom-air interfaces using PRESAGE^®^ 3D dosimeter and Monte Carlo simulations

**DOI:** 10.1088/1361-6560/aaaca2

**Published:** 2018-02-26

**Authors:** Filipa Costa, Simon J Doran, Ian M Hanson, Simeon Nill, Ilias Billas, David Shipley, Simon Duane, John Adamovics, Uwe Oelfke

**Affiliations:** 1 Joint Department of Physics, The Institute of Cancer Research and The Royal Marsden NHS Foundation Trust, London SM2 5NG, United Kingdom; 2 CRUK Cancer Imaging Centre, The Institute of Cancer Research, London SM2 5NG, United Kingdom; 3 Metrology for Medical Physics, National Physical Laboratory, Hampton Road, Teddington TW11 0LW, United Kingdom; 4 Department of Chemistry and Biology, Rider University, Lawrenceville, NJ 08648, United States of America; filipa.costa@icr.ac.uk

**Keywords:** 3D dosimetry, Monte Carlo, optical-CT, PRESAGE, MR-linac, quality assurance

## Abstract

Dosimetric quality assurance (QA) of the new Elekta Unity (MR-linac) will differ from the QA performed of a conventional linac due to the constant magnetic field, which creates an electron return effect (ERE). In this work we aim to validate PRESAGE^®^ dosimetry in a transverse magnetic field, and assess its use to validate the research version of the Monaco TPS of the MR-linac. Cylindrical samples of PRESAGE^®^ 3D dosimeter separated by an air gap were irradiated with a cobalt-60 unit, while placed between the poles of an electromagnet at 0.5 T and 1.5 T. This set-up was simulated in EGSnrc/Cavity Monte Carlo (MC) code and relative dose distributions were compared with measurements using 1D and 2D gamma criteria of 3% and 1.5 mm. The irradiation conditions were adapted for the MR-linac and compared with Monaco TPS simulations. Measured and EGSnrc/Cavity simulated profiles showed good agreement with a gamma passing rate of 99.9% for 0.5 T and 99.8% for 1.5 T. Measurements on the MR-linac also compared well with Monaco TPS simulations, with a gamma passing rate of 98.4% at 1.5 T. Results demonstrated that PRESAGE^®^ can accurately measure dose and detect the ERE, encouraging its use as a QA tool to validate the Monaco TPS of the MR-linac for clinically relevant dose distributions at tissue-air boundaries.

## 1. Introduction

The integration of magnetic resonance imaging (MRI) into a radiotherapy treatment platform provides images with high soft-tissue contrast that can be used to perform accurate image-guidance during treatment delivery, giving confidence to apply radiation with tight planning target volumes margins, and thus potencially reducing the toxicity risks and leading to higher tumour control. There are currently five separate designs for integrating an MRI scanner and a radiotherapy treatment device (Fallone *et al* 2009, Raaymakers *et al* 2009, Keall *et al* 2014, Mutic and Dempsey 2014, Mutic *et al* 2016). These designs differ in the method of radiation delivery (linear accelerator (linac) or cobalt teletherapy), the orientation of the main (**B0**) magnetic field (parallel or perpendicular to the radiation beam) and the magnetic field strength (0.35 T to 1.5 T). One of seven MR-linac prototypes (Elekta Unity, Elekta AB, Stockholm, Sweden), consisting of a modified 1.5 T MRI system (Philips, Best, The Netherlands) and a 7 MV linac (Elekta AB, Stockholm, Sweden) that rotates around a ring outside of the magnet (Lagendijk *et al* 2008, Raaymakers *et al* 2009), has been installed at our institute as part of the Elekta MR-linac Consortium (Kerkmeijer *et al* 2016).

Quality assurance (QA) performed in this MR-linac will differ from that required for a conventional linac due to the presence of the imaging **B0** field, which is perpendicular to the beam direction. The Lorentz force produced by this constant magnetic field deflects the paths of moving electrons, thus redistributing the absorbed dose, with some of these secondary electrons that would normally exit the tissue being directed back into it, leading to an electron return effect (ERE). The impact of the Lorentz force on the secondary electrons creates a reduced build-up distance and a strong dose increase at the proximal side of an air cavity and a reduction at its distal side (Raaijmakers *et al* 2004, 2005, Raaijmakers *et al* 2007a, 2007b).

Being able to have high resolution 3D dosimetry is of great interest for the increasingly sophisticated modern radiotherapy treatments, which often include high spatial dose modulation. Evidence is steadily accumulating to show the improved survival benefit of tight compliance radiation oncology delivery protocols of the actual delivery doses (Weber *et al* 2012, Dyk *et al* 2013).

The PRESAGE^®^ 3D dosimeter consists of a radiochromic plastic that shows an optical density change when irradiated and can thus be read out using an optical-CT scanner (Guo *et al* 2006). Contrary to other 3D dosimeters such as Fricke gels and polymer gels (Schreiner 2015), PRESAGE^®^ does not suffer from diffusion, or need an external container, making it a promising candidate to detect dose values at dosimeter-air interfaces. Previous work using large PRESAGE^®^ samples in an MR-guided radiotherapy context has shown good agreement with MC simulations, providing that spatial and temporal corrections are applied (Mein *et al* 2017, Rankine *et al* 2017). However, the critical issue of irradiating near tissue-air interfaces, where ERE can still occur even when opposing beams are used (Bol *et al* 2015), was not investigated.

This work is a demonstration of the use of PRESAGE^®^ for accurate relative dosimetry under the effect of a magnetic field to detect the ERE when air-gaps are present. We define a methodology by comparing PRESAGE^®^ measurements with EGSnrc MC simulations of an experimental set-up using an in-house phantom placed in an electromagnet and irradiated by a cobalt-60 (^60^Co) source. This methodology is of great interest to validate the research version of the Monaco (Elekta AB, Stockholm, Sweden) TPS which has been specially developed to model the magnetic field effect and dosimetry in the MR-linac (Bol *et al* 2012). We present the results of initial investigations in this area.

## 2. Material and methods

### 2.1. Experimental set-up

At the UK’s National Physical Laboratory (NPL, Teddington, UK) an electromagnet (250MM Electromagnet, GMW, USA) was installed adjacent to a (^60^Co) unit (Theratron 780C, Theratronics, Ontario, Canada), to study the effects of magnetic field (B) on dose distribution prior to the MR-linac coming into service.

**Figure 1.**
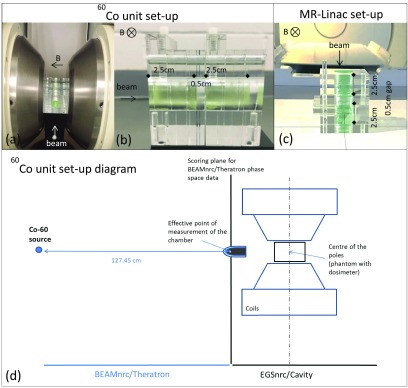
(a) Perspex phantom attached to the black Acetal base and positioned between the poles of the electromagnet (5 cm gap allow varying the magnetic field strength from 0 to 2 T). (b) PRESAGE^®^ sample configuration within the phantom to be placed between the electromagnet poles, at a distance of 162 cm from the ^60^Co source. (c) The same PRESAGE^®^ sample configuration within the phantom before its placement at the isocentre of the MR-linac. (d) EGSnrc MC simulation diagram of the ^60^Co unit set-up. Measurements were performed at 127.45 cm from the source, for convenience, and compared with BEAMnrc simulations. The output from BEAMnrc was saved as a phase space file and used as source input for EGSnrc/Cavity, where the electromagnet and the phantom geometry were created.

A Perspex phantom was developed, consisting of a number of separate 5 $\times$ 5 $\times$ 1 ${\rm cm}^{3}$ slabs that attach to each other and to the 5 $\times$ 7 ${\rm cm}^{2}$ surface of a black Acetal base, with the help of cylindrical rods. The phantom accommodates cylindrical samples of PRESAGE^®^ (Heuris Pharma, Skillman, NJ) of 2 cm diameter and is placed between the poles of the electromagnet as shown in (figure 1(a)).

### 2.2. PRESAGE^®^ 3D dosimeter / cuvettes irradiation

PRESAGE^®^ is a commercially available dosimeter which consists of a solid-state polyurethane matrix with a free radical initiator and a leuco-dye. When irradiated, PRESAGE^®^ undergoes an optical density (OD) change as the leuco-dye is oxidised from leuco malachite green to malachite green with a peak absorption maximum around 633 nm (Guo *et al* 2006). Two batches of custom-made cylindrical samples of PRESAGE^®^ (density 1.07 g⋅ ${\rm cm}^{-3}$) with 2 cm diameter and approximately 6 cm length were created with a plastic mould and cut to have 2.5 cm length (5 cm samples cut in half). For batch 1, the meniscus end of the samples was removed ($\leqslant$1 cm), while for batch 2, both ends of the samples were removed ($\leqslant$5 mm each end).

The reproducibility of our PRESAGE^®^ samples was determined via two irradiations, one using the cobalt source and one with an Elekta Synergy Linac (Elekta, Crawley, UK) with a 6 MV beam. On each platform, three sets of three cuvettes were irradiated in a water tank, with doses of 2, 6 and 10 Gy. Each sample was measured on four separate occasions (1, 2, 4 and 6 days) after irradiation, and the absorbance at 633 nm was recorded (an average of three consecutive readings) using a spectrophotometer (6705 Jenyway, Staffordshire, UK). For each radiation source, time point and each dose value, the mean and sample standard deviation for the three cuvettes were obtained and used to calculate a coefficient of variance.

The effect of magnetic field on the PRESAGE^®^ dose sensitivity was examined by irradiating cuvettes on the MR-linac under similar conditions except for the magnetic field. In the first case, the magnet was ramped down and in the second it was at field (1.5 T). Doses of 2.67, 6.23 and 9.79 Gy were given (300, 700 and 1100 MU). Two samples were irradiated at each combination of dose and magnetic field and the results read out on a Cary 50 Bio UV-VIS spectrophotometer (Agilent Technologies, California, USA), by recording a spectrum three times consecutively and taking the average of the points at 633 nm (the difference in readout equipment was dictated by equipment availability). Data were analysed by performing a linear fit on the absorbance values for the three dose points and using the LINEST function of Excel to estimate the sensitivity and its corresponding error from the gradient.

### 2.3. EGSnrc MC simulations in the presence of magnetic field

A source model for the ^60^Co unit has been previously implemented and validated at the NPL in BEAMnrc, an MC code based on EGSnrc (EGSnrc v4-r2-4-0) (Rogers *et al* 2005), for reference conditions (source to surface distance (SSD)  =  95 cm, 10 $\times$ 10 cm^2^ field). The ^60^Co unit comprises the ^60^Co source shielded by stainless steel and lead encapsulation, a tungsten collimator and 5 pairs of lead jaws that move together. The available source model was modified and a phase space file (ph-sp) was generated for non-reference conditions of SSD  =  127.45 cm, 10 $\times$ 10 cm^2^ field (Costa *et al* 2017).

To validate the BEAMnrc simulations, photon energy fluence spectra using the BEAMnrc utility code BEAMDP (Ma and Rogers 2016) were determined based on the ph-sp data and compared against measurements of air kerma. These profiles were obtained between  −15 cm and 15 cm in the $x$ and $y$ directions perpendicular to the beam, with an ionization chamber (Semiflex 31010, PTW, Freiburg, Germany) placed on an empty water tank. A schematic of the arangement for the measurements is shown on the diagram in figure 1(d). Gamma index analysis (Depuydt *et al* 2002), with 1.5% as dose difference (DD) and 1.5 mm as distance to agreement (DTA), was applied to assess the agreement between the measurements and the simulated photon energy fluence.

A second EGSnrc user code called Cavity, a C++ based MC system (Kawrakow *et al* 2009) was used to simulate the experimental set-up described in section 2.1. Both electromagnetic coils and the phantom were simulated and the ph-sp of the ^60^Co head was used as the input source (figure 1(d)). Simulations of two PRESAGE^®^ cylinders with their long axes parallel to the beam in the presence of different magnetic field strengths (0.5 T to 2 T) and different sizes air gaps (0.5 to 2 cm) were performed to study the influence of these parameters on the dose distribution. These simulations allowed us to identify the measurement conditions that would give us easily visible changes in the dose distribution, in particular in the ERE region, by only changing one parameter (air gap size or magnetic field strength). The lengths of the simulated PRESAGE^®^ samples were chosen based on the phantom length limitation (i.e. total for two halves of the phantom plus air gap) of 7 cm. One-dimensional profiles were obtained along the central region with variable sampling density, corresponding to the dose gradient along the beam direction (i.e. finer sampling where the dose changed more rapidly with distance). 0.5 $\times$ 1 $\times$ 1 mm^3^ and 1 $\times$ 1 $\times$ 1 mm^3^ voxels were used in the build-up region (first 4.5 mm of the sample); 2.5 $\times$ 1 $\times$ 1 mm^3^ voxels were used in the more slowly varying region distal to the depth of dose maximum; and 1 $\times$ 1 $\times$ 1 m^3^, followed by 0.5 $\times$ 1 $\times$ 1 mm^3^, in the steepening part of the curve in the edge region affected by the ERE (last 3 mm). Data were normalized to the centre of each simulated profile. The transport cut-offs were set to AE  =  ECUT  =  0.521 MeV for electrons, which corresponds to the rest mass plus the kinetic energy. The photons cut-offs were set to AP  =  PCUT  =  0.01 MeV. The macro provided by EGSnrc (emf_macros.mortran with EM ESTEPE  =  0.02) was included in the code to simulate magnetic fields (Kirkby *et al* 2008, Kawrakow *et al* 2016, Malkov and Rogers 2016), which were homogeneous and present through the simulation volume.

### 2.4. PRESAGE^®^ dose measurements in the presence of magnetic field

#### 2.4.1. PRESAGE^®^ irradiations versus EGSnrc MC simulations

On the basis of the simulation results, two sets of measurements were identified for subsequent experiments. Two sets of two PRESAGE^®^ samples from batch 1, separated by 0.5 cm air gap, were irradiated (3 Gy at the centre of the electromagnet poles, measured with an ionization chamber), at 0.5 T and 1.5 T magnetic field strengths (figure 1(b)). The choice of these two irradiations was based on the simulations performed in section 2.3, where the effect of the air gap size and magnetic field strengths was tested. The ^60^Co output value was determined with a NE2611(UK) ionization chamber based on Lillicrap *et al* (1990) guidelines.

Axial and sagittal 2D dose distributions were also simulated for comparison with measurements. The beam exit end of the PRESAGE samples was simulated to compare with measured data. The axial 2D distribution obtained with PRESAGE^®^ was averaged over the last 1 mm of the sample. Percentage depth dose (PDD) profiles were simulated independently with variable sampling from 0.5 $\times$ 1 $\times$ 1 mm^3^ to 2.5 $\times$ 1 $\times$ 1 mm^3^, performed in a similar manner as described in section 2.3. The statistical uncertainty of all simulations was within 2% for 2D data and 1% for 1D profiles. Measured and simulated profiles were normalized to the center of each simulated profile and compared using 1D gamma criterion analysis of 3% DD and 1.5 mm DTA (3%, 1.5 mm) (Depuydt *et al* 2002). 2D dose distributions were compared using 2D gamma analysis with the same criteria.

#### 2.4.2. PRESAGE^®^/optical-CT scanner

PRESAGE^®^ samples were imaged using an improved version of an in-house optical-CT microscopy scanner, which has previously been described and characterized for microbeam radiotherapy applications (Doran *et al* 2013). Improvements to the camera, computer and positioning system allowed faster and more accurate scans (McErlean *et al* 2016).

Optical-CT scanning was performed following the guidelines of Doran (2013a). Each sample was attached to the rotary sample holder and placed inside a glass tank with a matching liquid that has the same refractive index as PRESAGE^®^. A custom designed cap, developed in-house, was used to position PRESAGE^®^ cylinders within the sample holder. A small mark was made with a scalpel on both sample edges and the cap for reproducible positioning in pre and post-irradiation scans. As the positioning caps clamp onto the top 5 mm of the sample, it is not currently possible to image both distal and proximal ends of the sample simultaneously. Thus a pre-scan was performed for two separate regions of length 2.5 cm at opposite ends of the sample (i.e. the sample was inverted and then repositioned), allowing both end surfaces to be imaged. Each scan takes approximately 1 minute to obtain 1000 projections, each of 512 $\times$ 512 pixels, over 180$^\circ$ rotation for a field of view (FOV) of approximately (2.6 cm)^2^ to obtain a reconstructed image with a voxel size of (0.052 mm)^3^. Projections were reconstructed by filtered back-projection and the reconstructed images were post processed using Matlab^®^. The scans were assembled together and the high intensity values caused by artefacts within the sample were identified and replaced by the median of the surrounding pixel values. A median filter was also applied to smooth the images.

### 2.5. PRESAGE^®^ irradiation on the MR-linac versus Monaco TPS simulations

PRESAGE^®^ irradiations were also performed at the MR-linac. The perspex phantom was used again to accommodate two PRESAGE^®^ samples with 2.5 cm length separated by a 0.5 cm air gap. The phantom was placed so that the MR-linac isocentre was 1 cm downstream with respect to the incident radiation from the proximal face of the phantom, as shown in figure 1(c). Two irradiations were performed, using samples from batch 2, one at 0 T and the other at 1.5 T, with the 7MV beam of the MR-linac, gantry 0, a 20 $\times$ 20 cm^2^ field and 500MU. PRESAGE^®^ samples, from batch 2, were scanned as described in section 2.4.1. The experimental set-up was recreated in silico with CARPE DICOM (Elekta AB, Stockholm, Sweden) and simulated with 1 mm resolution and 1% uncertainty using the GPU-based Monte Carlo dose calculation algorithm (GPUMCD) in Monaco TPS. Again, measured and simulated profiles were normalized to the centre of each simulated profile.

## 3. Results

### 3.1. PRESAGE^®^ cuvettes irradiation

For the reproducibility experiment, the cobalt source samples gave a coefficient of variance with a mean over all times and doses of 1.8% and range of [0.9%, 4.1%], whilst the equivalent result for samples irradiated on the linac was 2.9% [0.4%, 5.0%]. For the experiment testing the effect of magnetic field on the PRESAGE^®^, the measured sensitivities with and without the magnetic field were (0.0425 $\pm$ 0.0017) ${\rm cm}^{-1}$⋅${\rm Gy}^{-1}$ and (0.0434 $\pm$ 0.0001) ${\rm cm}^{-1}$⋅${\rm Gy}^{-1}$, a difference of 2.1%. It should be noted that from the three data points measured, the error in the slope given by the LINEST algorithm will not be accurately determined.

### 3.2. EGSnrc model validation and EGSnrc MC simulation in the presence of magnetic field

**Figure 2.**
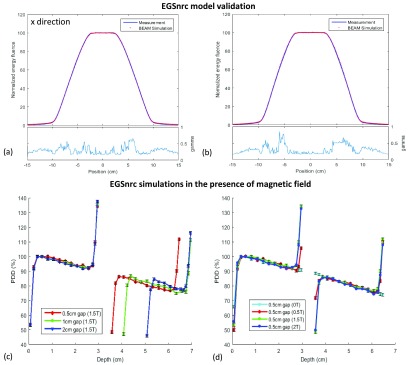
Comparison between air kerma measurements (Measurement) and simulated energy fluence profile (BEAM simulation) at 127.45 cm from the source, in (a) $x$ direction (horizontal) and (b) $y$ direction (vertical). Gamma values for 1.5%, 1.5 mm criterion are also shown. Simulated 1D profiles obtained by taking a central profile along the length of the two PRESAGE^®^ samples with different (c) air gap sizes (at 1.5 T) and (d) magnetic field strengths (with a 0.5 cm air gap). The simulated uncertainty is given by the error bars and was below 1% for each simulated data.

The phase space file validation of the source model is shown in figure 2(a) and (b). Profiles were normalized to the mean value of the central region, and showed good agreement with 100% points passing the gamma criterion of 1.5%, 1.5 mm.

Initial MC simulations of the experimental set-up showed that keeping an air gap of 0.5 cm and changing the magnetic field from 0.5 T to 1.5 T maximizes the differences between each PRESAGE^®^ sample (20% at the end of the first and second samples shown in figure 2(d)). This occurs as a 0.5 cm air gap is large enough for the electrons to curl back to the sample when a perpendicular 1.5 T magnetic field is present (radius  =  3.7 mm in vacuum), but less so when a 0.5 T (radius  =  11.2 mm in vacuum) is present instead. This justifies the selection of this set-up for the measurements described in section 2.4.1. The same does not happen for an air gap larger than 1 cm, as electrons will curve back for magnetic field strengths between 0.5 T and 2 T. At 1.5 T, changing the air gap size from 0.5 cm to 2 cm or more did not produce significant differences on the ERE values (2–3% on the first PRESAGE^®^ and 5–7% on the second) as shown in figure 2(c).

### 3.3. PRESAGE^®^ dose measurements in the presence of magnetic field

#### 3.3.1. PRESAGE^®^ irradiations versus EGSnrc MC simulations

**Figure 3.**
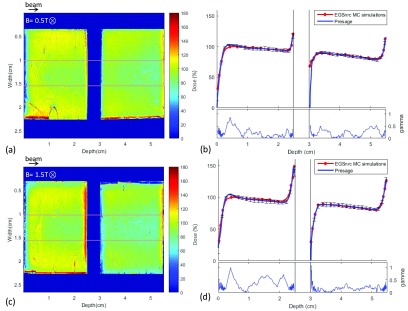
Normalized dose maps of the central sagittal slice along the two PRESAGE^®^ samples at (a) 0.5 T and (c) 1.5 T and respective (b) and (d) dose profile at the central region of interests (ROI) compared with simulated profiles. Error bars are representative of 1 standard deviation of the measured values within the ROI and account for readout artefacts and sample inhomogeneities. Gamma values for 3%, 1.5 mm gamma criterion are also shown.

Representative central slices from a sagittal view of the optical CT data are shown for two of the PRESAGE^®^ samples separated by an air gap of 0.5 cm and irradiated at 0.5 T and 1.5 T magnetic field strengths in figures 3(a) and (c) respectively. A number of image artefacts are present. These include microscopic inclusions in the PRESAGE^®^ samples and schlieren effects which are attributed to localised refractive index inhomogeneities (Doran 2009). Suspended particles in the matching liquid also degrade the image quality but are hard to remove completely in practice.

The normalized measured profiles in figures 3(b) and (d) were obtained from the regions of interest (ROI) shown in figures 3(a) and (c) respectively. Comparison with simulated profiles showed good agreement with a gamma passing rate (3%, 1.5 mm) of 99.9% for 0.5T and 99.8% for 1.5 T.

**Figure 4.**
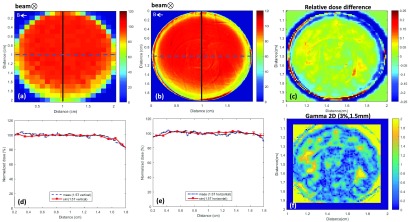
Normalized (a) simulated and (b) measured axial slice dose distribution of the last 1 mm of the first PRESAGE^®^ cylinder shown in figure 3(c). (c) Relative difference between measurements and EGSnrc MC simulations. (d) Vertical and (e) horizontal measured and simulated profiles excluding the last 1 mm edge. Error bars are representative of 1 standard deviation of the measured values within the ROI. (f) Gamma map for 3%, 1.5 mm of measured and simulated axial slice.

In figures 3(a) and (c) one can see that the dose increase due to the ERE is not uniform laterally within the last 2 mm of the beam exit area of each PRESAGE^®^ sample. The red region is not central but displaced upwards. This asymmetry is also evident in figures 4(a) and (b), where the simulated and measured last 1 mm axial slice of the first PRESAGE^®^ sample irradiated at 1.5 T are shown. This vertical shift is perpendicular to both beam and magnetic field, as the electron paths curve and deposit their energy at the top of the sample. This leads to a corresponding dose deficit at the bottom of the sample, and this is visualized quantitatively in the vertical profile of figure 4(d) for both simulated and experimental data. By contrast the horizontal profile in figure 4(e) shows no variation. Comparison between measured and simulated vertical and a horizontal profiles are shown in figures 4(d) and (e) respectively and these are discussed in further detail below. For a visual 2D dose comparison, both simulated and measured averaged 2D slices were resized to a 0.25 cm pixel size and their relative difference shown in figure 4(c). A 2D gamma analysis for 3%, 1.5 mm was also performed and the gamma map can be seen in figure 4(f).

#### 3.3.2. PRESAGE^®^ irradiation on the MR-linac versus Monaco TPS simulations

**Figure 5.**
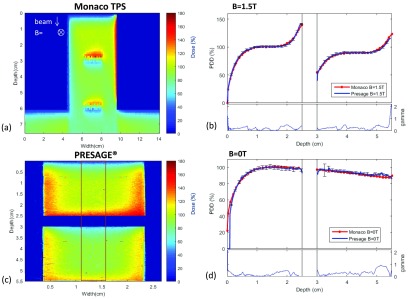
(a) Normalized dose distribution in the central sagittal plane simulated with Monaco TPS at 1.5 T. (c) Dose distribution obtained with PRESAGE^®^ samples irradiated at the same conditions. (b) Monaco and PRESAGE^®^ normalized dose profile at the central ROI at 1.5 T and (d) 0 T. Error bars are representative of 1 standard deviation of the measured values within the ROI. 1D gamma values for 3%, 1.5 mm gamma criterion are also shown.

Comparison between PRESAGE^®^ results and Monaco TPS simulation at the MR-linac are shown in figures 5(b) and (d), based on profiles along the length of both samples. A representative normalized dose distribution and central slices from a sagittal view are shown for both simulations (figure 5(a)) and Optical-CT data results with PRESAGE^®^ (figure 5(c)).

## 4. Discussion

The aim of this work was to perform a perliminary study of the effects of magnetic field on dose distributions as part of our methodological development prior to the MR-linac coming into service. PRESAGE^®^ has been used previously to measure highly conformal dose distributions in radiotherapy applications, showing accurate and reliable results to validate patient treatment plans before its delivery (Oldham *et al* 2008, Sakhalkar *et al* 2009, Brady *et al* 2010, Jackson *et al* 2015). PRESAGE^®^ also showed good potential to detect the ERE in the presence of cylindrical air cavities (Lee *et al* 2017a), but has not yet been assessed in the presence of air gaps and compared directly with MC simulations.

Previous published literature has reported a PRESAGE^®^ reproducibility of better than 2% (Guo *et al* 2006). It was not the original intention of this study to make definitive measures of absolute absorption, since many prior studies have demonstrated the difficulty of this task and the level of precautions necessary (Olding and Schreiner 2011, Skyt *et al* 2011). For our ERE measurements, it was the relative dosimetry within a single scan that was of interest. Thus, we did not attempt to control for day-to-day variations in temperature and other factors. Nor did we perform prior studies to establish the performance of the spectrophotometers as a gold standard. Instead, the results from the reproducibility experiment were used as a guide to determine whether the radiation sensitivities with and without magnetic field were significantly different. We observed that this difference was 2.1% and this is of the same order as the mean coefficient of variance in the reproducibility study. This suggests that the magnetic field does not affect the sensitivity, but, in view of other research in this area (Mathis *et al* 2014, Lee *et al* 2017b), further dedicated experiments would be needed to establish this conclusively.

Similar results were reported by others for doses up to 5 Gy (Lee *et al* 2017b), but for higher doses, sensitivity differences above 9% were obtained (Mathis *et al* 2014, Choi 2016). This difference might be explained by variations in the fromulation of the samples. Measured dose profiles in the presence of magnetic field showed generally very good agreement near dosimeter-air interfaces. In particular, the ^60^Co irradiations represent an extremely challenging test case, with the ERE that we wish to detect occurring over the last 3 mm of the sample (figures 3(b) and (d)). On the MR-linac itself, the range of the effect is closer to 1 cm and correspondingly less affected by optical artefacts (figure 5(b)).

These findings are very encouraging, because optical measurements have historically been extremely challenging at interfaces between media. There are two main reasons:

(i) The chemical manufacturing process can lead to sample inhomogeneities near surfaces, for example, through the effect of temperature during curation and, in the case of PRESAGE^®^ because of a loss of solvent from the dosimeter edges, leading to a concentration of various reactants. This can lead to a varying radiation sensitivity profile. Different radiation sensitivities within a PRESAGE^®^ sample have been reported before for large PRESAGE^®^ cylinders, showing 5% to 20% differences between the centre and the periphery, depending on the sample (Dekker *et al* 2016). We found similar non-uniformities in our samples, as exemplified by figures 3(a) and 5(c). The effect might be expected to be particularly pronounced in our small-diameter samples, where the surface-to-volume ratio is large. These effects may be responsible for the slight disagreement in the build-up region shown in the left-hand parts of the profiles in figures 3(c) and (d). The inhomogenities are also visible in figure 5, but the build-up shows better agreement, which could be due to the use of a different batch (batch 2), or the physical removal of both sample edges, whereas for batch 1, only the beam exit side of the samples was removed. A further batch-specific difficulty that we encountered during this work was the formation of surface nubs/bubbles on the PRESAGE^®^ due to low levels of moisture interacting at the interface between the mould surface and the curing polyurethane.
(ii) The difficulty of obtaining an exact match in optical index between a sample and the liquid in the optical CT scanner matching tank leads to a well known ring artefact (Doran 2013a). Most authors using larger samples have ignored this effect, but it occurs at exactly the position of interest in the profiles of figures 4(d) and (e). The effect can be modelled (Doran *et al* 2001), but since the shape of the roll off at the sample edge depends on both the refractive index and the dose-dependent sample absorption, correction requires detailed work to characterize each dosimeter batch. Although this is not an issue for this work, this effect will be investigated in detail before the use of these size dosimeters to validate more clinically relevant irradiations scenarios. Arranging the sample in such a way that the desired interface to measure is the one at the flat face of the dosimeter makes the task much easier. While we can still get surface artefacts within 1 mm of the samples surface, excellent results are obtained throughout the profiles as shown in figures 3 and 5.

For the above reasons, the comparison between EGSnrc MC simulation and experiment in figure 4(d) are not satisfactory over the edge region. However, we suggest that figure 4(e) provides early evidence of the ERE that matches simulation. Also important to note is the misalignment that might also have occurred between the pre and post-irradiation scan. As the OD is fairly homogeneous throughout the sample prior to irradiation, when the pre scan is subtracted from the post scan, small misalignments (less than 1$^\circ$ rotation about an axis perpendicular to the plane of the images in figures 3(a) and (b)) are not expected to influence the dose results. However, the existence of small imperfections inside and also at the surface of the PRESAGE^®^ samples results in more noise in the data if the pre and the post irradiation images are not well aligned. This effect, which was not quantified in this work, highlights the need for a more reproducible way to place the samples.

Measured and simulated PDD profiles on Monaco showed good agreement, with a gamma (3%, 1.5 mm) passing rate of 98.4% at 1.5 T and 99.6% at 0 T. This initial assessment based on profiles shows the potentialities of PRESAGE^®^ to detect the ERE accurately and to validate more complex dose distributions simulated with Monaco TPS.

## 5. Conclusion

While PRESAGE^®^ readout data can be improved by reducing edge and schliere artefacts, and a better understanding of the sample inhomogenities is still needed, good agreement between measured and computed results for both 1D profiles and 2D planes in the ERE region showed the great capabilities of PRESAGE^®^ to detect the ERE. This study gives encouragement for the use of PRESAGE^®^ on the MR-linac to validate Monaco TPS for more complex dose distributions near tissue-air interfaces.
